# Prostate Cancer Incidence in U.S. Counties and Low Levels of Arsenic in Drinking Water

**DOI:** 10.3390/ijerph17030960

**Published:** 2020-02-04

**Authors:** Jaeil Ahn, Isabella J. Boroje, Hamid Ferdosi, Zachary J. Kramer, Steven H. Lamm

**Affiliations:** 1 Department of Biostatistics, Bioinformatics, and Biomathematics, Georgetown University School of Medicine, Washington, DC 20007, USA; ja1030@georgetown.edu; 2 Center for Epidemiology and Environmental Health (CEOH, LLC), Washington, DC 20016, USA; Borojej@gmail.com (I.J.B.); Hamid@ceoh.com (H.F.); zkramer95@comcast.net (Z.J.K.); 3 Department of Epidemiology and Biostatistics, Milken Institute School of Public Health, George Washington University, Washington, DC 20052, USA; 4 Department of Global Health, Milken Institute School of Public Health, George Washington University, Washington, DC 20052, USA; 5 Department of Health Policy and Management, Bloomberg School of Public Health, Johns Hopkins University, Baltimore, MD 21205, USA; 6 Department of Pediatrics, Georgetown University School of Medicine, Washington, DC 20007, USA

**Keywords:** prostate cancer, low level arsenic exposure, drinking water, linear-quadratic model

## Abstract

*Background:* Although inorganic arsenic in drinking water at high levels (100s–1000s μg/L [ppb]) increases cancer risk (skin, bladder, lung, and possibly prostate), the evidence at lower levels is limited. *Methods*: We conducted an ecologic analysis of the dose-response relationship between prostate cancer incidence and low arsenic levels in drinking water in a large study of U.S. counties (*N* = 710). County arsenic levels were <200 ug/L with median <100 ug/L and dependency greater than 10%. Groundwater well usage, water arsenic levels, prostate cancer incidence rates (2009–2013), and co-variate data were obtained from various U.S. governmental agencies. Poisson and negative-binomial regression analyses and stratified analysis were performed. *Results*: The best fitting polynomial analysis yielded a J-shaped linear-quadratic model. Linear and quadratic terms were significant (*p* < 0.001) in the Poisson model, and the quadratic term was significant (*p* < 0.05) in the negative binomial model. This model indicated a decreasing risk of prostate cancer with increasing arsenic level in the low range and increasing risk above. *Conclusions*: This study of prostate cancer incidence in US counties with low levels of arsenic in their well-water arsenic levels finds a j-shaped model with decreasing risk at very low levels and increasing risk at higher levels.

## 1. Introduction

Arsenic is a well-known human carcinogen, primarily noted as an etiological agent for skin cancer, bladder cancer, and lung cancer. Occupational health studies have demonstrated this association through inhalation exposure of industrial particulates, and environmental health studies have demonstrated this association through ingestion exposure from drinking water. These findings have been shown for dosages in the range of 100 s to 1000 s of micrograms per day or per liter. The pattern of the arsenic dose-response curve at <100 μg/L is unclear. 

The evidence for exposures in the 1 s to 10 s of micrograms per day is less certain with studies showing slopes that are either significantly positive, significantly negative, or not significantly different from zero. The US drinking water standard for arsenic was first set at 50 μg/L as of 1942 by the United States Public Health Service (USPHS) and more recently at 10 μg/L as of 2006 by the United States Environmental Protections Agency (USEPA) [1].

Human cancers, other than skin, bladder, and lung cancers, have intermittently been found to be associated with arsenic exposure, including prostate cancer. The first report associating arsenic ingestion with prostate cancer was that of Chen and his colleagues in their mortality studies of internal cancers from the black foot disease (BFD) endemic areas of southwest Taiwan [2]. An increased risk of prostate cancer mortality was only observed for those with arsenic exposures greater than 300 μg/L in their drinking water (*RR* = 4) or with greater than 600 ug/L (*RR* = 6) but not for those with arsenic levels <300 μg/L (*RR* = 0.3) [2]. Additional studies from Taiwan also found increased prostate cancer risk but only among the residents of the high arsenic BFD-endemic areas of southwest Taiwan [3,4,5].

Studies outside of Taiwan reported prostate cancer associations at arsenic levels of 100–200 μg/L and at >200 μg/L in Australia [6] and at exposures in the 1000 s of μg/L-years [equivalent to 20 years at 50–250 + μg/L] in Utah [7]. In contrast, a Danish study reported no increased risk of prostate cancer incidence [*IRR* = 1.0] in Denmark where average exposures ranged between 0.05 and 25.3 μg/L (mean = 1.2 μg/L) [8].

In recent years, three studies have reported analyses of prostate cancer risk with low drinking water arsenic levels. A study of American Indian populations with exposure levels as high as 61 ug/L in public wells and greater than 50 μg/L in private wells showed increased prostate incidence for those in the top quintile of urinary arsenic levels [9]. A study from Illinois reported a significantly increased prostate cancer incidence for counties with mean arsenic levels in the top tercile (1.61–16.23 ug/L) [10], and a study from Iowa reported significantly increased prostate cancer incidences for counties with mean arsenic levels in the middle tercile (2.07–2.98 μg/L) and top terciles (2.99–18.6 μg/L) [11]. As the data for these studies were each aggregated into only three exposure strata, analyses for the shape of the dose-response relationship was limited.

We previously [12] examined the dose-response relationship between drinking water arsenic levels in U.S. counties and the incidence rate of lung cancer in the same counties, based on 757 US counties with recent (2009–2013) lung cancer incidence rates. In that analysis, the exposure data had been developed by the United States Geological Survey (USGS) and the cancer incidence rates had been developed by the National Cancer Institute (NCI). We now propose, in like manner, to use these same US national datasets to examine the dose-response relationship between drinking water arsenic level and prostate cancer incidence rates for US counties in the same respect.

## 2. Materials and Methods

This ecological study investigates the dose-response relationship between prostate cancer incidence and median drinking water arsenic levels (μg/L) from groundwater for U.S. counties. We have assessed the dose-response relationship between prostate cancer and arsenic levels in drinking water using county level exposure data aggregated by the United States Geological Survey (USGS) and cancer incidence data aggregated by the National Cancer Institute (NCI) and as previously described [12]. 

### 2.1. Arsenic Exposure Data

The National Water Information System (NWIS) exposure data provides the inorganic arsenic measurements (ug/L) for groundwater wells across the U.S. from the National Water-Quality Assessment program of the USGS. Hydride generation and inductive coupled plasma mass spectrometry (ICP/MS) were performed for the arsenic analyses. Made public in November of 2001, the National Water Information System (NWIS) dataset contained the most current inorganic arsenic measurements (μg/L) of groundwater wells in the U.S. [13]. The public use data set did not include the data in the governmental data set that had been embargoed at the state or county level. The sampling dates for these wells ranged from 1976 to 2001 with a median date of 1988, thus more than a 20-year latency for the cancers diagnosed in 2009–2013, a reasonable latency for solid cancers [14]. Based on which wells supplied each county, a median was calculated by the USGS using the arsenic data. Measurements below the limit of detection (LOD) (usually 1 μg/L) were entered into the data set as LOD/sqrt(2). Analyses were based on the data in the public use database. 

The median county arsenic level was used as the summary metric due to the limited number of wells within many of the counties. Using the median instead of the mean provided greater stability in countering potential high outlying measurements. Drinking water supplies with arsenic levels <50 μg/L constituted most (99%) of the data used to examine the dose-response between low levels of arsenic in drinking water and incidence rates of prostate cancer. Levels of 50 μg/L or greater were recorded for fewer than 5% of the counties.

USGS data showed the proportions of county residents that were dependent upon groundwater wells (either public or private) for their drinking water supply [15]. Complete data sets were available for 1985, 1990, and 1995. The average dependency rates for 1985, 1990, and 1995 were used as the estimates for county dependency.

As the purpose of this paper was to examine the dose-response relationship for prostate cancer incidence at low levels of arsenic exposure, the analytic data set was restricted to those counties (FIPS codes) where the maximum arsenic level was <200 μg/dL, the median and the mean were <100 ug/dL, and the dependency was 10% or greater. These are the same parameters that the US EPA [16] used in their examination of lung cancer incidence at low levels of arsenic exposure.

### 2.2. Alternative Carcinogenic Exposures

Other known environmental carcinogenic risk factors that entered the analyses were cigarette smoking and radon. Cigarette smoking entered the analyses as both the prevalence of current smokers and the prevalence of ex-smokers, using county-specific, gender-specific information for 2008-2010 obtained from the Small Area Estimates of the National Cancer Institute (NCI) [17]. Radon exposure entered the analyses as being counties identified by the USEPA as having predicted average indoor radon screening levels greater than the action level of 4 pCi/L [18].

### 2.3. Prostate Cancer Incidence Data

Age-adjusted county-specific prostate cancer incidence rates for 2009–2013 were obtained from the state cancer profiles derived by the National Cancer Institute [19]. Rates were based on the 2010 population data with the 2000 US standard population age-gender distribution as the reference population. Prostate cancer incidence rates were reported for the male population based on incidence cases over the five-year interval. Rates were suppressed by the state if the county had fewer than 16 prostate cancer cases over the five-year period, or if the data were prohibited from release by state administrative or legislative decision (Kansas, Minnesota, and Nevada).

### 2.4. Demographic Data

Demographic variables were acquired from the 2010 American Fact Finder site of the U.S. Census Bureau including race (White, Black Asian, Other), ethnicity (Hispanic), educational attainment (completion of high school or equivalency (5-year average)), poverty (proportion below poverty level), residency (proportion living in same county the previous year), and median household income (MHHI [$ K]) [20]. The race and ethnicity data were proportions from the 2010 U.S. census, while the data for the other variables were estimated 5-year averages (2009–2013). County-specific proportions of the 2010 county population that were not urban were obtained from the US Census geo urban area reference website. Obesity prevalence rates by sex were obtained from the CDC’s Diabetes Data and Trends site [21].

### 2.5. Geographical Mapping

County-specific data on groundwater dependency, water usage, and population served in each individual county were obtained from the United States Geological Survey (USGS). Water supply was categorized as either self-supplied domestic water (i.e., local private wells) or public supply sources, which were further categorized as being from groundwater or surface water. USGS data from 1985, 1990, and 1995 were used to develop county-specific data on total population, population using public water from groundwater, population using public water from surface waters, and population using self-supplied waters with county-specific summaries as the average of those from the three reports.

### 2.6. Statistical Analysis

Poisson regression models were used to examine the relationship between the median arsenic levels in wells used as a drinking water source for each county and the county’s prostate cancer incidence, as had been similarly performed for lung cancer incidence [22]. The Poisson regression model was formulated as follows:

log(*λc*) = log(*Nc*) + *β*0 + *β*1 × *Arsenic exposure_c_* + *γ^T^* × *Fc* (1)

where the natural logarithm of the expected number of prostate cancer incidence (*λc*) was the dependent variable and the median groundwater arsenic concentration was the primary independent variable with the county (FIPS) being the geographic aggregate of the data. The offset, log (*Nc*), was defined as the natural logarithm of the county male population (*Nc*). Covariates (Fc) include demographic variables such as race, socioeconomic status, education, and confounders such as groundwater dependency, smoking prevalence rates, radon, and obesity. Both Fc-unadjusted and Fc-adjusted models were fitted. The Akaike information criteria (AIC) and the Bayesian information criteria (BIC) were used to assess the best fitting model among linear, quadratic, and cubic expansions of the polynomial model. The model-fit diagnostics using residual analyses were performed to check the adequacy of the fitted models. Cook’s distance was used to identify the outliers and influential observations [23]. The sensitivity of the findings was assessed by tightening each of the model parameters in the Poisson regression models. To account for over-dispersion, a negative-binomial regression model was employed. Results with a two-sided *p* < 0.05 (Wald |*Z*-score| > 1.96) were considered to be statistically significant. Poisson regression analyses were additionally run with arsenic exposures expressed as quartiles of the median arsenic levels. A stratified analysis was also run. 

## 3. Results

**Figure 1:**
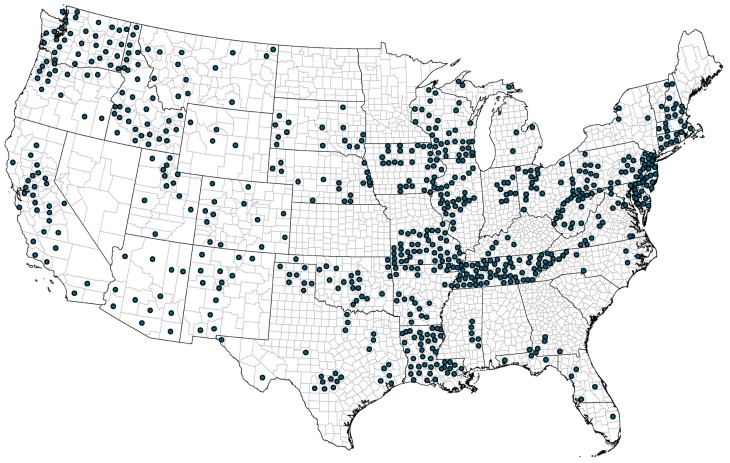
Map of U.S. Counties the in Arsenic and Prostate Cancer Study.

The original USGS data set included 868 U.S. counties with arsenic levels for groundwater wells used for drinking water of which 742 also had prostate cancer incidence rates from the NCI and of which 737 had maximum arsenic level <200 μg/L, 741 had median <100, and 721 had dependency greater than 10%. The primary analytic model for the examination of the dose-response relationship between low-level arsenic exposure in drinking water from groundwater sources and the incidence of prostate cancer was the Poisson linear-quadratic model with restrictions that the maximum arsenic exposure be <200 μg/L, the median arsenic exposure be <100 μg/L, and the dependency be greater than 0.100 (i.e., >10%). These criteria were met by 715 counties (FIPS areas). The male population of these 715 counties comprised 34% of the U.S. 2010 male population (51 M/152 *M* = 34%) and 257 million-person years of observation. Figure 1 below demonstrates that these 715 counties were in 41 states and distributed throughout the 48 contiguous states.

### 3.1. Location

The counties in these analyses come from 41 of the 50 U.S. states, generally distributed across the 48 contiguous states with particular frequency in the states of Tennessee and New Jersey (Figure 1). Other areas of increased prevalence seem to be in the Pacific Northwest (Washington, Idaho, and Oregon) and in the Mississippi River valley (Wisconsin, Illinois, Iowa, Missouri, and Louisiana). None of the counties in Nevada, North Dakota, Minnesota, Kansas, Maine, Hawaii or Alaska are in the study. These states are not in the study either because their data were embargoed or because none of their counties met the criteria for the study.

### 3.2. Population Characteristics

The data characteristics of the analytic variables for the 715 counties in the analysis are seen in Table 1 for the county male populations.

#### 3.2.1. Outcome

Age-adjusted prostate cancer incidence rates ranged between 44.8 and 220.3 cases per 100,000 male residents per year and varied only by a five-fold factor. The median and mean rates differed little at 116.4 and 117.9 per 100,000, respectively. The number of prostate cancers per county for the five-year period varied markedly, ranging from 10 to 12,652. This reflected the marked range in male populations for the various counties, from about 1700 to about 2 million. The estimated number of new prostate cancer cases in the five-year period for male residents of the county was the outcome variable in the analyses.

#### 3.2.2. Exposure

The levels of arsenic in the drinking water were the principle exposure measure of interest. These counties were predominantly dependent on these groundwater wells for their drinking water supply with a median dependency of 87% and a mean dependency of 76%. The range was from 10% to 100%. The number of such wells per county ranged from 1 to 190 with a median of 2 and a mean of 8.1. Most counties had only one or two public wells (376/715 = 52%) as their drinking water source.

Three metrics of drinking water arsenic exposure at the county level were obtained –median, minimum, and maximum. The arsenic levels ranged from non-detected (limit of detection [LOD] of 1 μg/L) to 190 μg/L. For data analysis, specimens with no detected arsenic were entered as 0.7071 ug/L, i.e., 1/(sqrt(2)) x LOD. The county median arsenic levels ranged between non-detected (i.e., 0.7) and 52.5 μg/L with a median of 0.9 and a mean of 2.1. The county minimum arsenic levels ranged from non-detected to 42 ug/L with a median of 0.7 and a mean of 1.3. The county maximum arsenic levels ranged from non-detected to 190 μg/L with a median of 0.9 and a mean of 7.96. The larger differences between means and medians generally reflect the disparate distribution of the underlying data.

#### 3.2.3. Demographics

Data characteristics of the analytic co-variates for the individual counties – median, mean, minimum, and maximum – are also shown in Table 1. For the analytic co-variates, the means and the medians are generally similar, suggesting that they were relatively symmetrically distributed. The mean and median prevalence of current smokers was about 25% and that of ex-smokers was about 30%. The obesity prevalence was about 30% and ranged from 15% to 40%. The prevalence of residents with less than a high school education was about 84% with a range of 59% to 97%. The proportion with no recent change in residency (i.e., did not change county of resident within the past year) was high (0.94 with range of 0.78–0.98). Most counties had populations with about 15% at or below the poverty line (range 3–48%) and had mean or median household incomes of about $45 K (range 23.9–106.1). The proportion rural ranged from 0 to 100% with a mean or median of about 50%.

The size of the counties, in terms of male population, varied widely from a minimum of <1700 to a maximum of >2,000,000. That the median of the populations (19,977) is only about a quarter of the mean of the populations (71,914) indicates that most of the counties in this study had small populations. This suggests that most large cities received their drinking water supply from surface sources rather than from underground sources.

The county populations varied widely in their racial characteristics. While the median and mean proportion white was about 90%, the white population ranged between 20% and 99% of the male population. The median and mean proportion black was 2% and 7%, respectively, and the range was from 0% to 69%. The mean being about four times higher than the median indicated that most counties had very few blacks, and the analogous data also show that they had very few Hispanics, Asians, or Others. The males of “other” racial group were most likely American Indian, as most of the counties with > 25% “other” were in Arizona, New Mexico, or Oklahoma.

### 3.3. Poisson Regression Models

Poisson regression analyses were fitted with the restrictions that the maximum exposure be <200 ug/L, that the median exposure be <100 ug/L, and that the dependency be >0.100 (i.e., >10%). A Goodness-of-fit was examined for outlier and influential counties, and three were identified. They each included a large city, generally near the U.S.-Mexico border, [El Paso in El Paso County, Texas; San Antonio in Bexar County, Texas; and Phoenix in Maricopa County, Arizona]. The unadjusted analysis included data on 715 counties before exclusion of the outliers and on 712 counties afterwards. The adjusted analysis included data on 710 counties before exclusion of the outliers and on 707 counties afterwards. The linear model showed a poor goodness of fit with an increasingly non-symmetrical residual pattern. 

Higher-order polynomial models were then sequentially run as quadratic and cubic models. The linear-quadratic model was found to be a better fit for the Poisson regression than was the linear model, based on the AIC and BIC goodness-of-fit criteria, as well as on the residual analysis, and to be a borderline better model than the cubic model. The linear-quadratic model was also found on the basis of the AIC and BIC goodness of fit criteria to be a better fit that the model using quartiles of the median arsenic exposures.

The models, both unadjusted and adjusted, were first run with all counties and with all examined co-variates, and then without the outliers and both with and without the non-significant covariates (Table 2).

The primary finding is that the dose-response relationship between the prostate cancer incidence and the median water arsenic level for low level arsenic levels was that of a linear-quadratic model with a statistically significant negative coefficient (slope) for the linear term and a statistically significant positive coefficient for the quadratic term. This held true also with the exclusion of outliers, both with and without the non-significant co-variates. This yields a J-shaped curve with a prostate cancer risk decreasing from about <1 ug/L to about 8–18 ug/L and then increasing towards 50 ug/L. The best point estimate of the minimum risk level is 11 ug/L, based on −1/2 the ratio of the coefficients of the linear term and the quadratic term of the adjusted model with exclusion of the outliers.

### 3.4. Sensitivity Analyses

The above Poisson analyses have used the restrictions of maximum arsenic <200 ug/L, median <100 ug/L, and groundwater dependency >0.10 (10%). Each of these restrictions can be tested for their sensitivities. Table 3 shows the adjusted Poisson regression models of the prostate cancer incidence with removal of the outliers and of the non-significant co-variates when the maximum arsenic level is reduced from <200 ug/L to <100 ug/L, as well as when the median arsenic level is reduced from < 100 ug/L to <50 ug/L and when the groundwater dependency is narrowed from >10% to > 80%.

In each case, the data had an excellent fit to the Poisson linear-quadratic model with a statistically significant positive coefficient for the quadratic term of the median arsenic level and usually a statistically significant negative coefficient for the linear term of the median arsenic level.

In addition to testing the sensitivity of the analysis to the extreme value restrictions we have used in the Poisson regression analyses, we have also tested the sensitivity of the analysis to the assumptions of the Poisson model. The Poisson model assumes that the mean and the variance are equivalent, which is very restrictive in real applications. We assess this assumption by fitting the negative-binomial regression model that accounts for over-dispersion. 

Table 4 compares the findings of the Poisson and the Negative-Binomial regression models given the same restrictions of maximum <200 ug/L, median <100 ug/L, and groundwater dependency >10%. The comparison in Table 4 is between the analytic results for 710 counties using both the adjusted Poisson model and of the adjusted negative-binomial model.

Both the Poisson regression analysis and the negative-binomial regression analysis show comparable results with similar coefficients. Statistical significance was less in the negative-binomial analysis due to the inflated standard errors caused by over-dispersion. This indicates that statistical significance for a linear-quadratic curve remains valid even after correction for over-dispersion. Under the negative binomial analysis, the goodness-of-fit statistics also show that the linear-quadratic model outperforms both the linear and the cubic models. 

### 3.5. Stratified Analysis

**Figure 2:**
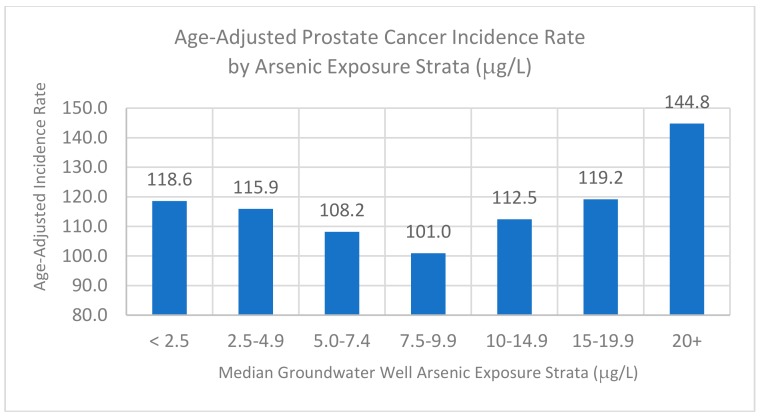
Stratified Analysis of Age-Adjusted Prostate Cancer Incidence Rate by Median Groundwater Well Arsenic Exposure Strata for US Counties.

The above analyses have sought a model with a continuous function across the exposure range. A second approach has been to develop a stratified analysis of the data. Figure 2 presents a stratified analysis of the prostate cancer data (age-adjusted incidence rates) by strata of the median groundwater well arsenic levels of the various counties in the study. The bin sizes are narrow (2.5 μg/L) at the low end of the exposure range where the data are quite dense, wider (5 μg/L) in the mid-range, and then open ended at the high end.

The stratified analysis reveals a similar pattern to the regression analysis with a decline in the risk as the arsenic exposure increases toward 10 ug/L and then an incline as it increases to about 50 ug/L. As with the Poisson regression model, the stratified model indicated that the cancer risk increased from the minimum risk, whether the arsenic exposure level decreased or increased. 

## 4. Discussion

Arsenic is a well-known human carcinogen as particularly well-demonstrated for skin, bladder, and lung cancer and is less well demonstrated for other cancers, including prostate cancer. The carcinogenicity of arsenic through the ingestion of arsenic in drinking water has been compellingly reported from exposures in the hundreds and thousands of micrograms (ug) per liter. At lower levels the association is less certain and shows different fits to either a linear model or a linear-quadratic model. Therefore, the pattern that carcinogenic risk follows for low levels of arsenic is uncertain. For instance, in the case of lung cancer, one report found a statistically significantly positive linear association [16], another found a statistically significantly negative linear associations [12], and a third found the best significant fit to be to a linear-quadratic model that had a statistically significant negative linear term and a statistically significant positive quadratic term [22]. The later report [22] was a systematic review and meta-regression analysis that showed for lung cancer a J-shaped model for the studies whose data went from low-level to high-level doses and found this pattern for each study design-ecological, case-control, and cohort. This study now shows for a second cancer, for prostate cancer, a J-shaped curve with respect to arsenic levels in the community water supply. 

For the prostate cancer incident rates, the best fitting model (based on AIC and BIC criteria) was the linear-quadratic model with a statistically significant negative linear term and a statistically significant positive quadratic term. This model presents a J-shaped model with a decreasing cancer rate as arsenic level increases from <1 μg/L to about 8–18 μg/L and then shows a change in direction with an increasing cancer rate as arsenic level increases towards 50 μg/L. This suggests that the minimum risk level for prostate cancer is an arsenic exposure of about 8–18 μg/L. This pattern has previously been observed for arsenic and bladder cancer and for arsenic and lung cancer and may be explained by different toxicological effects at lower and higher arsenic exposure levels (see below). 

### 4.1. Epidemiological Studies

The early studies from Southwest Taiwan associating arsenic with prostate cancer [2,3,4,5] were all with arsenic levels in the drinking water being many hundreds to thousands of ug arsenic per liter. The later, most recent studies [9,10,11], each had their top exposures in the range of 20–60 μg/L and reported increased risks of prostate cancer at their top arsenic exposure strata. However, unlike this study, they have not had sufficient data points to examine the dose-response relationship over their exposure range for low levels of arsenic.

This study, with data from more than 700 U.S. counties, has examined the dose-response relationship between low levels (median < 100 μg/L) of arsenic in the drinking water of U.S. counties and the incidence of prostate cancer. It has been found that the best fit is to a linear-quadratic model with a negative linear term and a positive quadratic term.

### 4.2. Arsenic metabolism

Drinking water is an oxygen-rich environment and inorganic arsenic in drinking water is thus primarily in the pentavalent form (As^V^) with some in the trivalent form (As^III^). The two forms can be interconverted within the body [24,25]. The reduction of pentavalent arsenate (As^V^) to trivalent arsenite (As^III^) occurs in the gastrointestinal tract by intestinal flora and in the blood and the liver by the enzyme arsenate reductase. Arsenite (As^III^) can then be detoxified through oxidative methylation to monomethylarsenate (MMA^V^) [also called monomethylarsonous acid] [26]. This occurs in the testes, kidney, and lungs by the enzyme arsenic (As^III^) methyltransferase [As3mt]. The cycle is repeated with the reduction of monomethylarsenate (MMA^V^) to monomethylarsenite (MMA^III^) and the oxidative methylation to dimethylarsenate (DMA^V^), followed by the reduction to dimethylarsenite (DMA^III^). The pentavalent forms (MMA^V^ and DMA^V^) are more water-soluble than the trivalent forms (MMA^III^ and DMA^III^) and, thus, are most commonly found in the urine. While most arsenic excretion is through the kidneys as urination, a lesser amount is excreted through the feces and in the keratin [27]. Arsenic concentrations by valence are not available in the groundwater arsenic data. At present, there are no studies that examine the valance state (III or V) of the arsenic exposure for the arsenic-associated cancers. 

### 4.3. Arsenic Toxicology

The trivalent methylated metabolites tend to be more cytotoxic than the inorganic trivalent arsenic itself [28]. There is a broad range of tissue concentrations that cause cytotoxicity in animal studies. Trivalent arsenic tends to show cytotoxicity at tissue concentrations in the μM range (> 0.1–10.0 μM (7.5–750 μg/L) [29], while pentavalent methylated arsenic is only toxic in the mM range (i.e., at >75 mg/L or >75,000 μg/L) [30]. Thus, a cellular concentration of >0.1 ug/L (7.5 ug/L) has been considered to be a reasonable estimate for the minimum cellular concentration required for obtaining a biological effect, which may be either adaptive or toxic [31]. Effects of arsenic at low concentrations on the order of 0.1 uM (7.5 ug/L) appear to be adaptive, while concentrations above 1 uM (75 ug/L) are clearly cytotoxic [32]. Urinary levels of >0.1 uM (>7.5 ug/L) trivalent arsenic in humans are estimated to be associated with drinking water arsenic levels of 50–100 ug/L [31]. The threshold for potentially adverse cellular effects from exposure to inorganic arsenic in drinking water is thought likely to occur at urinary concentrations of trivalent arsenic above 0.2 um (15 ug/L), which corresponds to drinking water total arsenic concentrations above 65 ug/L [33].

Trivalent arsenic interacts directly with sulfhydryl-containing proteins, such as keratin in the skin, thus increasing its local concentration [34]. Since the binding of trivalent arsenic to the sulfhydryl units of proteins tends to deactivate them, a threshold effect may be observed whereby only higher concentrations of arsenic may lead to inhibition of metabolic processes. 

### 4.4. Carcinogenicity Studies

Long-term single-generation rodent bioassay studies have not demonstrated arsenic carcinogenicity, and arsenic-associated cancers are not found in nature, other than in humans. Rather studies from the laboratory [35,36,37,38,39,40] of Waalkes and Tokar have demonstrated transplacental carcinogenicity in mice, primarily in the lung and liver. There are no reports examining for prostate cancer. 

Waalkes and his colleagues have published a series of studies that demonstrate transplacental carcinogenicity in mice from arsenic exposure. His earlier one-generation studies [35,36,37,38] with arsenite exposures in the 42.5–85 ppm range in the maternal generation variably found either lung cancers or liver cancers in the offspring. Their initial two-generation study [39] had maternal and off-spring exposure to arsenite at 6, 12, and 24 ppm range reported dose-related cancers at multiple sites (lung adenocarcinoma, liver, and uterus). Their subsequent two-generation study [40] had exposures up to 5 ppm (5000 ppb) with intermediary exposures at 50 and 500 ppb. Lung cancers were only in excess in the 50 ppb dose group and not in either the 500 ppb or 5000 ppb group. These results appear to fit a bimodal model but not a linear no-threshold model. 

### 4.5. Mode of Action 

The mode of action for arsenic carcinogenicity is under discussion, as arsenic does not act directly with DNA (deoxyribonucleic acid) [41]. The leading theory on the mode of action for arsenic carcinogenicity is that of the induction of cytotoxicity with consequential regenerative proliferation [42]. This has been most clearly demonstrated in the DMA^V^ (dimethylarsinic acid) bladder cancer model in rats [43]. Cohen first pointed out the importance of regeneration in human carcinogenesis in 1991 [44]. It has now been estimated that the threshold level for arsenic cytotoxicity in humans from arsenic exposure in drinking water is approximately 50–100 μg/L with higher levels for regenerative proliferation [42]. Our data are consistent with the Cohen model.

Arsenic toxicity mechanisms differ at low and high doses with autophagy inhibition occurring at low in-vitro doses (18–150 ppb) and oxidative stress occurring at high in-vitro doses (800 ppb) [45]. A review of arsenic-mediated activation of the NRF2 transcription factor antioxidant pathway has found that activation of Nrf2 both protects and contributes to arsenic toxicity and carcinogenicity [46]. In studies in the ppm range, the genomic analysis of the urothelium in animals exposed to inorganic arsenic has shown an initial change corresponding to cytotoxicity after two weeks of exposure with genomic changes indicative of a proliferative response appearing after twelve weeks of exposure [47].

With regard to prostate cancer, in vitro study of human prostate epithelial cells has demonstrated that oxidative DNA damage as evidenced by biomethylation may lead to arsenite-induced malignant transformation of human prostate epithelial cells with chronic high arsenic exposure, i.e., continuously exposed for 29 weeks to 5 μM (375 μg/L) arsenite [48,49]. While in-vitro studies have thus demonstrated that prostate epithelial cells have the potential to undergo malignant transformation, *in-vivo* studies have not demonstrated an increased frequency of prostate cancers [26]. 

### 4.6. Dose-Response Models

In human epidemiological studies, arsenic exposures in the range of 100–200 μg/L appear to be the threshold for increased cancer rates [42]. The epidemiological literature on environmental arsenic carcinogenicity contains studies showing both linear and J-shaped curves. An arsenic and skin cancer study from Inner Mongolia, China, showed a hockey-stick distribution with a threshold of 122 μg/L [50]. A 2005 analysis of the one-well villages in SW Taiwan showed a negative slope for bladder cancer mortality in the range of 10–126 μg/L and a positive slope in the range of 250–550 μg/L, an early indication of a J-shaped curve in environmental arsenic carcinogenicity [51]. A 2015 systematic review and analysis of lung cancer studies with exposures above and below this level found an increased rate only at exposures above 136 μg/L with a range of 97–206 μg/L, depending on study design [22]. It further revealed that the data fit a dose-response linear-quadratic model with both a statistically significant negative linear term and a statistically significant positive quadratic term. It was interesting to note that that pattern was observed independent of study design. The same dose-response pattern was observed for ecological studies and for non-ecological studies, both case-control and cohort. That pattern for lung cancer is quite consistent with what has been found in this dose-response ecological analysis of prostate cancer incidence and exposure to lower levels of arsenic in drinking water. 

This linear-quadratic model yields a J-shaped curve which has been suggested to reflect the induction of anti-carcinogenic processes at low levels and of pro-carcinogenic processes at higher levels with the transition between the two yielding a J-shaped curve [22]. This may reflect the over-stimulation of repair or protective mechanisms at low levels of exposure that then become overwhelmed by pro-carcinogenic mechanisms at higher levels. This may also relate to the Cohen explanatory model with cytotoxic effects at low or early toxicity exposure levels and proliferative effects at high or later toxicity exposures [30,42,44].

### 4.7. Strengths and Limitations

The strengths of this study include that the data are for over 700 U.S. counties and have been collected and validated by government agencies in their normal course of operations and independent of this study. These data are in the public domain. This is the largest study undertaken for low-level arsenic exposure and prostate cancer incidence. The study includes over 700 independent counties, from over 80% of U.S. states, with over 1/3 of the U.S. male population, and over 250 million person-years of observation. 

The limitations of this study are primarily those intrinsic to an ecological study, that the analytic unit is the county, usually with data presented as aggregate data (mean or prevalence), rather than the individual, with individualized data. The major limitations of the drinking water arsenic exposure estimate include that most counties have fewer than three ground water sources of drinking water, that most of the measurements are quite low, and that the proportion of residents using the water sources is variable. Further, the arsenic levels in this study are in the range equivalent to the intake of arsenic from foods. That intake includes both inorganic arsenic and organic arsenicals, such as arsenobetaine from fish. We have no county-specific information on the daily intake of arsenic through the diet.

## 5. Conclusions

This is an ecological study of prostate cancer cases (2009–2013) in U.S. counties that receive their drinking water from ground water sources having low levels of arsenic (median < 100 μg/L). This includes more than 700 counties, in more than 80% of the U.S. states, and with more than 250 million person-years of observation of the male population. Data for outcome, exposure, and co-variables came from U.S. governmental agencies. 

Both Poisson regression and negative-binomial regression were conducted with the best-fitting models being a linear-quadratic model. The Poisson model was a linear-quadratic model with a negative linear term and a positive quadratic term. The quadratic term was statistically significantly positive in both the Poisson and negative-binomial models, and the linear term was statistically significantly negative in the Poisson model. Both the Poisson analysis and the stratified analysis presented J-shaped models. 

This study of prostate cancer incidence in U.S. counties with low levels of arsenic in their well-water arsenic levels finds a J-shaped model with decreasing risk at very low levels and increasing risk at higher levels. This is similar to the J-shaped curve that had previously been shown for lung cancer. 

## Figures and Tables

**Table 1:** Data Characteristics of Analytic Variables for 715 Individual Counties.

*Variable*	Median	Mean	Minimum	Maximum
Outcome				
Prostate Cancer Rate (per 100,000)	116.4	117.9	44.8	220.3
Count (5-year estimate)	111	442	10	12,652
Exposure				
Dependency	87%	76%	10%	100%
Well Count	2	8.1	1	190
As Median (ug/L)	0.9	2.1	0.7	52.5
As Minimum (μg/L)	0.7	1.3	0.7	42
As Maximum (μg/L)	1.0	7.96	0.7	190
Co-Variates				
Current Smoker (%)	25.6	25.3	9.54	40.0
Ex-Smoker (%)	29.6	29.9	15.9	48.3
Obesity (%)	31.9	31.2	15.1	41.2
Education (<HS) (%)	84.3	83.0	58.6	97.4
Residency (Same County) (%)	94	93	78	98
Poverty (%)	14	15	3	48
Income ($K)	44.8	47.3	23.9	106.1
Rural (%)	48	49	0	100
*Population*				
Male Population	19,977	71,914	1,698	2,037,405
Hispanic (%)	4	9	0	80
White (%)	92	87	20	99
Black (%)	2	7	0	69
Asian (%)	1	1	0	22
Other (%)	2	4	1	79

**Table 2:** Unadjusted and Adjusted Poisson Regression Models of Median Arsenic Exposure and Prostate Cancer Incidence (Maximum < 200 μg/L; Median < 100 μg/L, and Dependency > 10%).

Unadjusted	Exposure Range for Median Arsenic Level		
	All	Without Outliers	Without Non-Significants ^+^
Unadjusted Median Model			
N	715	712	712
Arsenic^2^	0.0007 ***	0.0005 ***	0.0005 ***
Arsenic	−0.0310 ***	−0.0225 ***	−0.0225 ***
Intercept	−5.0460 ***	−5.0480 ***	−5.0480 ***
**Adjusted**	**All**	**Without Outliers**	**Without Non-Significants ^+^**
Adjusted Median Model			
N	710	707	707
Arsenic^2^	0.0003 ***	0.0002 ***	0.0002 ***
Arsenic	−0.0105 ***	−0.0044 **	−0.0043 **
GW Dependency	0.0243 ***	0.0204 **	0.0196 **
Current Smoker	−0.0031 ***	−0.0019 **	−0.0192 **
Ex-Smoker	0.0016 *	0.0002	-
Radon (>4 pCi/L)	−0.0229 ***	−0.0380 ***	−0.02869 ***
Obesity	−0.0044 ***	−0.0028 ***	−0.0029 ***
Education (<HS grad)	−0.0023 ***	−0.0001	-
Residency (Same cnty)	0.5992 ***	0.9458 ***	0.9353 ***
Income (MHHI $K)	0.0001	0.0006	−0.8308 ***
Poverty	−0.9332 ***	−0.8308 ***	-
Rural	−0.0976 ***	−0.0972 ***	−0.0952 ***
Hispanic	−0.2762 ***	0.7371 ***	0.2969 ***
Black	0.7872 ***	−0.2438 ***	−0.7414 ***
Asian	0.1804 ***	0.370	-
Other	−0.1993 ***	−0.1950 ***	−0.1988 ***
Intercept	−5.1643 ***	−5.7424 ***	−5.7519 ***

^+^ Excludes both outliers and non-significant covariates. * *p* < 0.05; ** *p* < 0.01; *** *p* < 0.001.

**Table 3:** Adjusted Poisson Regression Models ^+^ of Median Arsenic Exposure and Prostate Cancer Incidence with Maximum <100 ug/L, Median <50 ug/L or Dependency >80%.

Variable	Maximum ^+^ <100 μg/L	Median ^+^ <50 μg/L	Dependency > 80 μg/L
N	695	707	392
Arsenic^2^	0.0001 **	0.0002 ***	0.0003 ***
Arsenic	−0.0015	−0.0044 **	−0.0090 ***
GW Dependency	0.0354 ***	0.0204 **	−0.3562 ***
Current Smoker	−0.0010	−0.0019 **	−0.0026 **
Ex-Smoker	−0.0010	−0.0002	−0.00465 ***
Radon (>4 pCi/L)	−0.0397 ***	−0.0380 ***	−0.0491 ***
Obesity	−0.0040	−0.0028 ***	−0.0046 ***
Education (<HS grad)	0.0029 ***	−0.0001	0.0038 ***
Residency (same cnty)	1.1929 ***	0.9458 ***	1.4419 ***
Income (MHHI $K)	−0.0004	0.0006	0.0017 **
Poverty	−0.7191 ***	−0.8193 ***	−0.6423 ***
Rural	−0.0813 ***	−0.0972 ***	−0.0966 ***
Hispanic	−0.1367 ***	0.2371 ***	−0.4040 ***
Black	0.7731 ***	0.7438 ***	0.5714 ***
Asian	−0.0387	−0.0370	0.5339 ***
Other	−0.2432 ***	−0.1950 **	−0.4291 ***
Intercept	−6.2642 ***	−5.7423 ***	−5.6349 ***

^+^ Outliers excluded. * *p* < 0.05; ** *p* < 0.01; *** *p* < 0.001.

**Table 4:** Adjusted Poisson Regression and Negative-Binomial Regression Models of Median Arsenic Exposure and Prostate Cancer Incidence (Maximum < 200 μg/L; Median < 100 μg/L, and Dependency >10%).

Regression Model	Poisson	Negative-Binomial
Adjusted Median Model		
N	710	710
Arsenic^2^	0.0003 ***	0.0002 *
Arsenic	−0.0105 ***	−0.0050
GW Dependency	0.0243 ***	0.0432
Current Smoker	−0.0031 ***	−0.0068 **
Ex-Smoker	0.0016 *	−0.0014
Radon (>4 pCi/L)	−0.0229 ***	0.0086
Obesity	−0.0044 ***	−0.0003
Education (<HS grad)	−0.0023 ***	0.0011
Residency (same cnty)	0.5992 ***	0.7427 **
Income (MHHI $K)	0.0001	−0.0005
Poverty	−0.9332 ***	−0.7564 **
Rural	−0.0976 ***	−0.1123 **
Hispanic	-0.0262 ***	−0.2405 *
Black	0.7872 ***	0.7987 ***
Asian	0.1804 ***	−0.0654
Other	−0.1993 ***	−0.1028
Intercept	−5.1643 ***	−5.5708 ***

* *p* < 0.05; ** *p* < 0.01; *** *p* < 0.001.
